# The relationship between cognition and white matter tract damage after mild traumatic brain injury in a premorbidly healthy, hospitalised adult cohort during the post-acute period

**DOI:** 10.3389/fneur.2023.1278908

**Published:** 2023-10-23

**Authors:** Jacqueline F. I. Anderson, Lucy E. Oehr, Jian Chen, Jerome J. Maller, Marc L. Seal, Joseph Yuan-Mou Yang

**Affiliations:** ^1^Melbourne School of Psychological Sciences, The University of Melbourne, Parkville, VIC, Australia; ^2^Department of Psychology, The Alfred Hospital, Melbourne, VIC, Australia; ^3^Developmental Imaging, Murdoch Children's Research Institute, Melbourne, VIC, Australia; ^4^General Electric Healthcare, Melbourne, VIC, Australia; ^5^Monash Alfred Psychiatry Research Centre, Melbourne, VIC, Australia; ^6^Department of Paediatrics, The University of Melbourne, Parkville, VIC, Australia; ^7^Neuroscience Research, Murdoch Children's Research Institute, Melbourne, VIC, Australia; ^8^Department of Neurosurgery, Neuroscience Advanced Clinical Imaging Service (NACIS), The Royal Children's Hospital, Melbourne, VIC, Australia

**Keywords:** mild traumatic brain injury, cognition, white matter tract, diffusion tensor imaging, neurite orientation dispersion and density imaging (NODDI)

## Abstract

**Introduction:**

Recent developments in neuroimaging techniques enable increasingly sensitive consideration of the cognitive impact of damage to white matter tract (WMT) microstructural organisation after mild traumatic brain injury (mTBI).

**Objective:**

This study investigated the relationship between WMT microstructural properties and cognitive performance.

**Participants, setting and design:**

Using an observational design, a group of 26 premorbidly healthy adults with mTBI and a group of 20 premorbidly healthy trauma control (TC) participants who were well-matched on age, sex, premorbid functioning and a range of physical, psychological and trauma-related variables, were recruited following hospital admission for traumatic injury.

**Main measures:**

All participants underwent comprehensive unblinded neuropsychological examination and structural neuroimaging as outpatients 6–10 weeks after injury. Neuropsychological examination included measures of speed of processing, attention, memory, executive function, affective state, pain, fatigue and self-reported outcome. The WMT microstructural properties were estimated using both diffusion tensor imaging (DTI) and neurite orientation dispersion and density imaging (NODDI) modelling techniques. Tract properties were compared between the corpus callosum, inferior longitudinal fasciculus, uncinate fasciculus, anterior corona radiata and three segmented sections of the superior longitudinal fasciculus.

**Results:**

For the TC group, in all investigated tracts, with the exception of the uncinate fasciculus, two DTI metrics (fractional anisotropy and apparent diffusion coefficient) and one NODDI metric (intra-cellular volume fraction) revealed expected predictive linear relationships between extent of WMT microstructural organisation and processing speed, memory and executive function. The mTBI group showed a strikingly different pattern relative to the TC group, with no relationships evident between WMT microstructural organisation and cognition on most tracts.

**Conclusion:**

These findings indicate that the predictive relationship that normally exists in adults between WMT microstructural organisation and cognition, is significantly disrupted 6–10 weeks after mTBI and suggests that WMT microstructural organisation and cognitive function have disparate recovery trajectories.

## Introduction

Mild traumatic brain injury (mTBI) that results in hospital treatment, occurs in at least 100–300/100,000 individuals per year (1). Cognition is routinely affected by mTBI in the acute period, most commonly in the domains of speed of information processing, attention, memory and executive function (1). Full resolution of objective cognitive difficulties occurs for the majority of individuals within 3 months of injury (2). During normal recovery, however, as well as for the 20% who do not recover within the typical timeframe (1), cognitive impairment is a substantial contributor to poor psychosocial outcome and disability after mTBI (3–5).

In healthy adult populations, objective cognitive performance has been shown to be associated with WMT microstructural organisation, as measured with diffusion-tensor imaging (DTI) metrics. Specifically, fractional anisotropy (FA), which quantifies degrees of diffusion directionality, is positively associated with general cognition (6, 7), speed of processing (6), attention (6), memory (8) and executive function (6); mean diffusivity (MD), which quantifies magnitude of diffusivity, is negatively associated with processing speed (6) and executive function (6). Within the mTBI population, a recent meta-analysis of hospitalised mixed-mechanism adults found that structural white matter tract (WMT) changes are also associated with cognitive changes (9). Specifically, WMT pathology is associated with dysfunction in attention, memory and executive function, with poorer performance in these cognitive domains significantly associated with reduced FA. Poorer performance in the domains of attention and memory are also associated with increased MD. Despite being commonly affected by mTBI, processing speed has not been included in sufficient DTI studies to enable a meta-analysis.

Although there is currently no consensus regarding whether specific tracts are associated with deficits in specific cognitive domains post mTBI, it has been established that WMT damage after mTBI typically occurs in long-range association and interhemispheric tracts (10). These include the corpus callosum (CC), superior longitudinal fasciculus (SLF), inferior longitudinal fasciculus (ILF), uncinate fasciculus (UF) and anterior corona radiata (ACR) (10–13). In particular, DTI metrics most typically demonstrate reduced FA and increased apparent diffusivity coefficient (ADC; considered broadly synonymous to MD, as MD is the mean ADC of the diffusion tensor) in these tracts after mTBI; these have been interpreted as representative of diffuse axonal injury (10).

Changes in DTI metrics are multi-determined, being influenced by many white matter microstructural properties that are directly affected by mTBI-related pathological mechanisms, including dysmyelination, axonal loss, and/or reduced axonal diameters (14, 15). The coherence of axonal fibre arrangements, a microstructural factor potentially unrelated to the brain injury, also influences DTI metrics (15–17). Importantly, DTI cannot model multiple axonal fibre orientations (“crossing fibres”) with a single MRI voxel (18), which is problematic given they are present in over 90% of brain white matter regions (19).

Neurite orientation dispersion and density imaging (NODDI) (20) is a biophysical diffusion weighted imaging (DWI) model that provides more specific information about WMT microstructural properties than DTI. The NODDI model fits the DWI signal into three assumed tissue compartments: the intra-neurite, extra-neurite and cerebrospinal fluid compartments. Two useful quantitative metrics derived from NODDI are the intra-cellular volume fraction (ICVF), which is a measure of neurite density, and the orientation dispersion index (ODI), which quantifies the bending and fanning of neurites (20). The metric ODI has a strong negative correlation with FA, whereas ICVF has a weak positive correlation with FA (20, 21).

It has been shown that NODDI is sensitive to mTBI-related WMT changes (13, 22–25). Indeed, the current cohort of mTBI participants has been shown to have WMT microstructural disorganisation relative to the current TC sample, which is evident on both DTI and NODDI metrics (13). Few studies have investigated the relationship between NODDI metrics and measures of cognition, however. One study showed linear relationships between ICVF and measures of attention, memory and executive function (25), but the direction of these linear relationships was reversed when mTBI participants’ performances were compared with a non-mTBI trauma control (TC) group. Individuals with mTBI showed negative linear relationships with measures of attention, memory and executive function. The researchers did not assess processing speed and examined individuals approximately 2 weeks after injury, when injury-related physiological fluctuations are still present and substantial recovery is ongoing; this makes cognitive function more variable and limits reliability of examination (26, 27). The study also used tract based spatial statistics (TBSS), which relies on a whole brain analysis of WMTs that have been normalised prior to analysis. This approach may not be appropriate for identifying subtle changes in WMT structure (28). A second study found ODI was positively correlated with processing speed in a combined mTBI and healthy control group, but also used TBSS and examined individuals with mTBI even earlier - less than 7 days after injury (29).

The aim of the current study was to investigate the relationship between cognitive performance and DTI- and NODDI-derived WMT microstructural metrics of individual tracts, in the post-acute period (>6 weeks) after mTBI. It was hypothesised that both the mTBI and TC groups would demonstrate positive linear associations between FA and cognitive performance in all cognitive domains, and both groups would demonstrate a negative linear association between ADC and processing speed, attention and memory. In contrast, it was hypothesised that the mTBI group would demonstrate negative linear associations between ICVF and attention, memory and executive function, whereas the TC group would demonstrate linear associations in the opposite direction on these variables. Finally, it was hypothesised that the TC group would demonstrate negative linear relationships between ODI and all cognitive domains, whereas the mTBI group would demonstrate positive linear associations between ODI and processing speed.

## Method

### Participants

Participants comprised individuals, excluding professional athletes and war veterans, who had suffered any traumatic injury (systemic and/or head) between September 2015 and April 2018, and been consecutively admitted to The Alfred hospital, Melbourne, Australia, in the preceding 6–12 weeks. Detailed description of the recruitment process and the recruitment decision tree have been reported previously (30, 31). All admitted trauma patients were approached for recruitment consideration. The mTBI group comprised 26 premorbidly healthy adults (22 male) aged 18–60 years (Mean = 34.81, SD = 13.76) whose traumatic injury included a head strike and fulfilled criteria for a mTBI event as defined by the World Health Organisation criteria (32), which can be summarised as (i) 1 or more of: confusion or disorientation, loss of consciousness for 30 min or less, post-traumatic amnesia less than 24 h, and/or other transient neurological abnormalities not requiring surgery; (ii) Glasgow Coma Scale score of 13–15 after 30 min or later upon presentation for healthcare. Exclusion criteria were individuals with: any previous neurological history; any history of heavy alcohol consumption (>5 standard drinks/day), intravenous or regular Class A drug use; any current Class A drug use; history of any significant psychiatric disorder and any current/recent diagnosis or treatment of depression and/or anxiety and/or post-traumatic stress disorder; current TBI (at time of hospital admission) as a result of physical assault/attack; and lack of conversational English fluency. The TC participants comprised 20 premorbidly healthy adults (18 male) aged 18–60 years (Mean = 38.75, SD = 12.59) whose traumatic injury had not included a head strike and who did not report any symptoms of mTBI; this group had the same exclusion criteria as the mTBI group, with the addition of having no previous head injury. No ethnic group differences existed. All participants provided informed consent and the project was approved by The Alfred hospital Human Research Ethics Committee.

### Measures

#### Premorbid cognitive functioning

The Wechsler Test of Adult Reading (WTAR) (33) is a word reading task, from which accurate estimates of premorbid intellectual functioning (PreIQ) can be derived in individuals with mTBI (34).

#### Processing speed

The Symbol Digit Modality Test – (SDMT) is a measure of processing speed that is sensitive to cognitive impairment after mTBI (35). It requires individuals to provide the correct number that corresponds to a given symbol, according to a reference key at the top of the page. On this version of the SDMT, the final score was number of correct items within 2 min.

#### PS/attention index

The Trail Making Test Parts A and B (TMTA and B) (36) measure processing speed and high level attention, respectively. These tests have high reliability and validity for mTBI populations (37, 38). The Victoria Stroop Dots trial (Stroop Dots) is a measure of processing speed (39). The Digit Span subtest from the Wechsler Adult Intelligence Scale – 4th is a valid, reliable and widely-used (40). Digit Span Total (DSp) is a global measure of attention; raw scores rather than aged scaled scores were used to enable comparative analyses with other cognitive measures.

#### Memory index

The Rey Auditory Verbal Learning Test (RAVLT) (41) is a reliable and valid measure of verbal memory (42). The total number of items learned on the 5 list learning trials (Total) assessed acquisition and the number of items recalled independently after a 25-min delay (Delay) assessed recall. The *Rey-Osterrieth Complex Figure Test* Delay (RCFT Delay) score (43) is a widely used measure of visual memory function that has good test–retest reliability (0.89) (44) and has been used with mTBI populations previously (45, 46). Ability to independently draw the stimulus figure after a 10-min delay was used as the measure of visual memory (RCFT Delay). All RCFT figures were scored by a single researcher, and intra-rater reliability was high (0.94).

#### Executive function index

The ratio of TMTB/TMTA was used as a measure of mental flexibility. The interference ratio of colour words/dots from the Victoria Stroop test (Stroop Int) is a measure of inhibitory control. Both of these measures have been shown to be sensitive to executive dysfunction after mTBI (37, 38, 47, 48).

#### Pain

The Short-Form McGill Pain Questionnaire (MPQ) (49) was used to measure pain. The MPQ requires respondents to endorse whether they have experienced different types (descriptions) of pain, and to what level, during the past week. It has excellent reliability and validity and has been used with mTBI populations (50).

#### Fatigue

The Multidimensional Fatigue Inventory (MFI) (51) was used to measure fatigue. The MFI is a 20-item questionnaire that measures five different types of fatigue and sums these together to provide a general measure of fatigue. It has good internal consistency (Cronbach’s alpha >0.076) and has previously been used in the TBI population (52, 53).

#### Quality of life

The RAND 36-item Short Form Survey (SF-36) (54) was used as a measure of general health related quality of life. This quality of life measure has been shown to be both reliable and valid in TBI populations (55).

#### Post-concussion symptoms

The Rivermead Post Concussion Symptoms Questionnaire (RPQ) is a widely used measure of post-concussion symptomatology. It assesses physical (10 items), psychological (3 items) and cognitive (3 items) symptoms experienced during the past 24 h (56). It has been shown to be elevated after mTBI and also other conditions (57–59).

#### Psychological distress index

Three widely used, valid and reliable questionnaires of psychological distress were used: The Inventory of Depressive Symptomatology (IDS) measures severity of overall depression (60). The Beck Anxiety Inventory (BAI) measures anxiety symptomatology (61). The PTSD Checklist for the DSM-5 (PCL-5) (62) measures the 20 symptoms of PTSD defined by DSM-5 (63). A single Psychological Distress Index (PDI) was created from standardised performances on the IDS, BAI and PCL-5. Specifically, responses on each measure were converted to a standardised 4-point scale and then summed together, resulting in a single variable comprising equivalent numbers of items measuring depression (IDS) and anxiety-based symptomatology (BAI and PCL-5).

Principal component analysis (PCA) was conducted on the cognitive measures to reduce the number of measures and identify a coherent group of cognitive indices in a statistically recommended manner (64). This analysis was conducted using a larger sample of mTBI and TC participants (*n* = 87), which had been recruited in an identical manner but had not undergone neuroimaging. As the SDMT was found to correlate moderately with most variables, it was removed and analysed separately. From the remaining variables, a three-component solution was supported, which explained 70.93% of the total variance. These components were interpreted as measuring Processing Speed/Attention, Memory and Executive Function. The variable to sample size ratio was 9:1, which compensates for a smaller sample size (*n* = 87) (65). The structure matrix of the PCA is presented in Supplementary Table S1.

Raw scores from test performances were converted to z-scores also using the aforementioned larger sample of mTBI and TC participants (*n* = 87). Index scores were then determined for each individual by averaging the z-scores for the tests comprising each index.

#### Measure of effort

The Digit Span (DSp) subtest from the Wechsler Adult Intelligence Scale, 4^th^ Edition (WAIS-IV) (40) was used as a measure of effort (66). Participants were identified as having problematic effort on testing if they failed on the subscales of Longest Digits Forward (Fail = 4 or less) and Longest Digits Backward (Fail = 2 or less) (66).

### Procedure

Following recruitment on the ward, within 1–4 days of injury, participants returned to the hospital for neuropsychological examination and MRI scans, conducted on the same day, 6–10 weeks after injury. Neuropsychological measures were conducted in the following sequence for all participants: SDMT, WTAR, Stroop, RAVLT, DSp, RCFT, TMT, RPQ, SF-36, MFI, IDS, BAI, PCL-5 and MPQ.

#### MRI data acquisition

MRI data (PRISMA3T Siemens, Erlangen, Germany) was acquired at the Baker IDI Heart and Diabetes Institute, Melbourne, Australia. High resolution volumetric T1-weighted images were acquired using the magnetisation prepared rapid gradient-echo (MPRAGE) sequence (240 × 256 mm acquisition matrix; FOV = 256 mm; 176 contiguous slices; 1mm^3^ isotropic voxel size; TR/TE = 2300/2.96 ms). The DWI data was acquired with multiband accelerated EPI sequences for multishell acquisition (128 × 128 mm acquisition matrix; FOV =256 mm; 75 contiguous slices; 64 *b* = 1,000 s/mm^2^ volumes, 64 *b* = 3,000 s/mm^2^ volumes, and 4 *b* = 0 s/mm^2^ volumes; 2mm^3^ isotropic resolution; TR/TE = 4800/88 ms; MB factor = 3). Additional volumes of reversed phase encoded b0 images were obtained to correct for susceptibility-induced geometric distortion (67).

#### MRI data processing

All DWI data and tractography reconstructions were processed using the MRtrix3 software package (version 0.3.16; Brain Research Institute, Melbourne, Australia^1^) with FSL functions (FMRIB’s Software Library^2^) incorporated in MRtrix3 command line usage. The NODDI model fitting and parametric maps were estimated using the NODDI Matlab Toolbox (Version 1.0.1). The DWI data was pre-processed to correct for thermal noise (68), gibbs-ringing artifacts (69), eddy current and motion artifacts (67, 70), susceptibility-induced geometric distortions (67), and *β1* bias field inhomogeneities (71). Local WM fibre-orientation distributions were estimated based on multi-shell multi-tissue Constrained Spherical Deconvolution (msmt-CSD) (72). Tractography, representing the CC, and the six pairs of long-ranged association WMTs or WMT segmented components, were reconstructed from each participant, using a probabilistic tracking algorithm (73), retained 2,500 streamlines per WMT, a FOD cutoff = 0.1 and other default tracking parameters, and manually placed regions of interest (ROI) in areas with known anatomical priors based on modifications made from previous published methods (74). The DTI and NODDI model fittings and associated parametric maps were estimated based on the subset of *b = 1,000 s/mm^2^* data and the multishell DWI data, respectively. Tract-wise averaged DTI and NODDI parametric estimations were then computed from binarised WMT masks derived from each tractography reconstruction.

**Figure 1:**
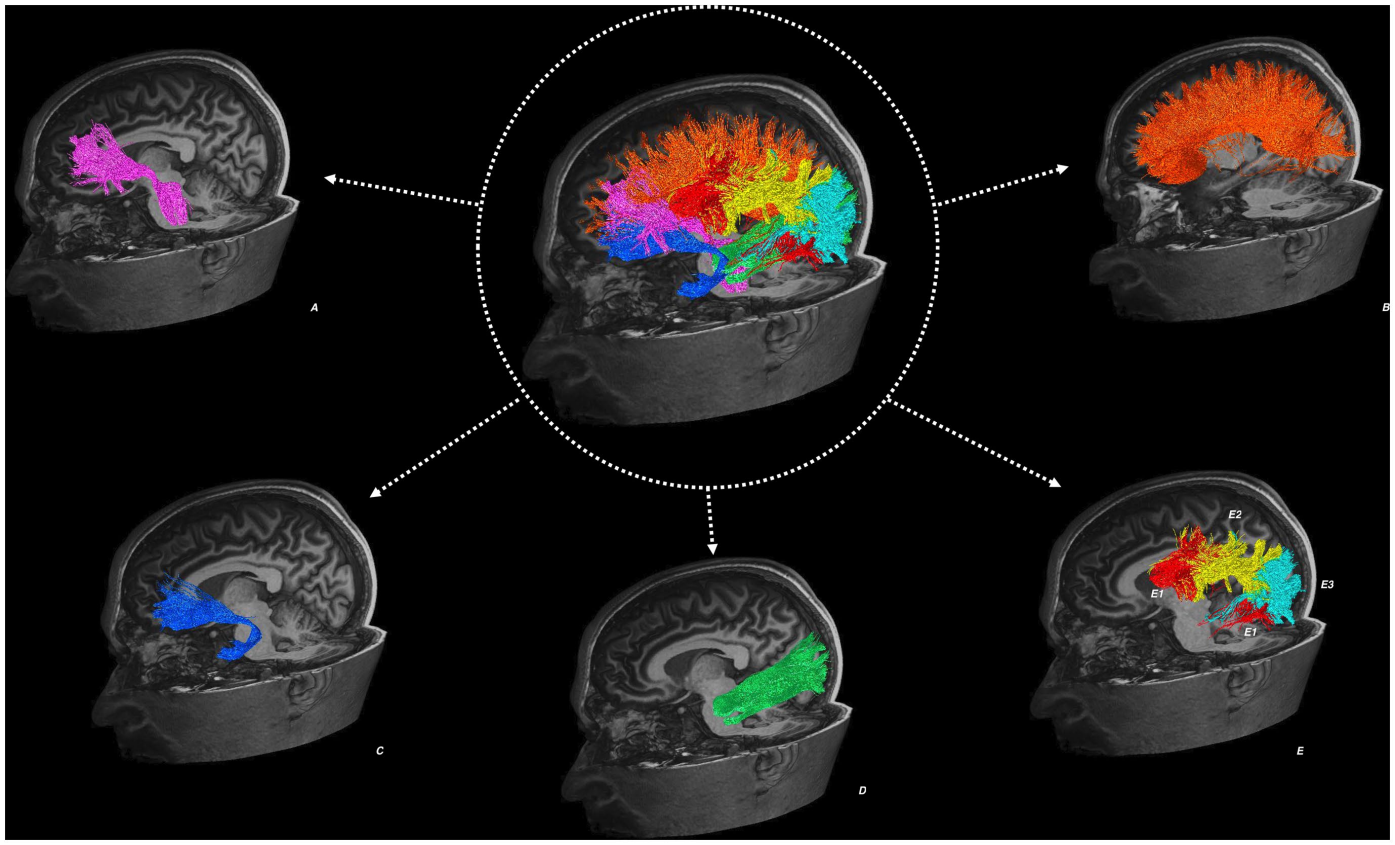
Tractography reconstructions from an example study participant. Showing the left anterior corona radiata (A), corpus callosum (B), uncinate fasciculus (C), inferior longitudinal fasciculus (D), superior longitudinal fasciculus (E) in three segmented components: the direct segment (E1), anterior indirect segment (E2), and posterior indirect segment (E3).

Figure 1 illustrates the reconstructed tractography images from a representative study participant.

### Statistical analysis

Statistical analyses were undertaken using Statistical Package for Social Sciences (SPSS version 26) software. Most demographic variables had a non-normal distribution, so Mann Whitney U tests were conducted to compare groups on these variables; the MPQ, MFI, SF-36 and days between injury and assessment were normally distributed enabling t-test analyses. Multivariate analyses of covariance (MANCOVA) were used to examine group differences on the cognitive variables, controlling for days post injury. Multiple regression analysis was conducted to identify group differences in the relationship between independent and cognitive variables. For significant interactions, additional regression analyses were undertaken separately for each group to examine whether DTI (FA, ADC) and NODDI (ICVF, ODI) metrics, from each WMT of interest, significantly predicted performance on cognitive testing. As research has demonstrated significant associations between DTI metrics and both age and predicted FSIQ (7, 75), these variables were included in the model as covariates. All analyses were re-run including sex as a co-variate. As this had no impact on the findings, we excluded this co-variate from the reported findings as increasing co-variates reduces the power of analyses. Consistent with modern statistical practise (76) in the context of using multiple univariate neuroimaging analyses, results were interpreted by identifying consistent overall patterns of findings and considering magnitudes of difference through inspection of confidence intervals, rather than focussing on specific *p*-values. A consistent overall pattern of results, which have confidence intervals that are not close to including 0.00, as we have in this paper, is considered to indicate a real effect (76).

## Results

The demographic details and injury characteristics for each group are presented in Table 1. No individual in either group failed the measure of effort. The between group comparisons of the DWI data have been previously published, with between group differences evident on DTI and NODDI metrics on all white matter tracts (13).

**Table 1:** Demographic and injury variables for mTBI and TC groups.

	TC (*n* = 20) *M (SD)*	mTBI (*n* = 26) *M (SD)*	*p*
*Demographics*			
Age (yrs)	38.750 (12.590)	34.808 (13.755)	0.272
Gender (% *F*)	10	15	0.601
Education (years)	13.581 (2.470)	14.432 (3.451)	0.460
PreIQ	106.050 (10.560)	106.192 (10.568)	0.938
MPQ	1.364 (0.999)	1.147 (0.988)	0.467
MFI	52.000 (12.057)	47.769 (13.515)	0.276
SF-36	77.000 (14.815)	76.200 (16.327)	0.865
RPQ	9.050 (8.457)	9.960 (7.835)	0.681
Psych Distress	1.137 (0.897)	1.171 (0.880)	0.713
*Injury-related variables (%)*			
MVA	20.00	7.69	
MBA	30.00	19.23	
Cycling accident	20.00	26.92	
Fall	20.00	19.23	
Sports-related	5.00	3.85	
Other	5.00	23.08	
GCS		14.5 (0.71)	
LOC (%<5 min)		81.77	
PTA (%<60 min)		50	
Inj to Ax (days)	49.950 (9.185)	63.269 (12.127)	**<0.001**

PreIQ, Predicted Full scale IQ; MPQ, Short-Form McGill Pain Questionnaire; MFI, Multidimensional Fatigue Inventory; SF-36, RAND SF-36 health related quality of life; RPQ, Rivermead post-concussion symptom questionnaire; Psych Distress, Psychological Distress Index; Other, Primarily pedestrian injuries from being hit by a vehicle, e.g., bicycle, tram, car; MVA, Motor vehicle accident; MBA, Motorbike accident; GCS, Glasgow Coma Scale score; LOC, Loss of consciousness; PTA, Post-traumatic amnesia; Inj to Ax, Days between injury and assessment.

The groups were well matched on all demographic variables and reported similar levels of post-concussion symptoms and quality of life, but the mTBI group had an average of 2–3 weeks of additional recovery time between injury and examination relative to the TC group. As between group comparisons were not carried out for the primary analyses of interest (regression analyses), this group difference was not included as a co-variate.

Analysis of the cognitive variables revealed no group differences on either the SDMT or cognitive indices (*p* > 0.225), as shown in Table 2. Effect sizes were small (0.01) to moderate (0.07) for all comparisons. T-test analyses indicated that the mTBI (*p* = 0.001) and TC (*p* = 0.002) groups were significantly slower on the SDMT than a previously reported healthy control sample ($\bar{X}=77.26,SD$ = 13.61) (77).

**Table 2:** Group comparison of cognitive variables.

Cognitive variable	TC (*n* = 20) *M (SD)*	mTBI (*n* = 26) *M (SD)*	*p*	Partial *η^2^*
SDMT (#correct)	64.500 (17.144)	66.308 (12.992)	0.571	0.026
Attention Index	−0.211 (0.980)	0.163 (0.462)	0.226	0.067
Memory Index	−0.236 (0.943)	0.181 (0.705)	0.242	0.064
Exec. Funct. Index	0.046 (0.753)	−0.037 (0.705)	0.933	0.003

SDMT, Symbol Digit Modalities Test; Exec. Funct., Executive Function.

After controlling for age and predicted FSIQ, a series of planned significant regression analyses revealed the groups repeatedly differed with respect to the relationship between the independent variables and the measure of cognition. Those models that were not significant revealed no relationship between the independent variables and cognitive measures for either group. Detailed results of the significant regression models, and subsequent regression analyses that identified a statistically independent relationship between an imaging metric and a cognitive variable, are presented in the Supplementary Tables S2–S6. Table 3 presents an overview, for each WMT, of those models that were significant overall (*p* < 0.05), *and* also revealed a significant independent relationship between one or more DWI metrics and the cognitive variable.

**Table 3:** Regression models with significant independent neuroimaging predictors by cognitive variables for each group.

Tract		SDMT			SP/Attention Index		Memory Index				EF Index		
	Metric	TC		mTBI	TC	mTBI	TC		mTBI		TC	mTBI	
CC	DTI	FA*	-ADC				FA	-ADC					
	*NODDI*	*ICVF*											
SLFR-ds			-ADC*					-ADC					
		*ICVF*											*-ICVF*
R-ai		FA	-ADC					-ADC					
									
R-pi			-ADC*					-ADC					
		*ICVF*					ICVF*						*-ICVF*
L-ds		FA	-ADC					-ADC				-ADC	-FA
		*ICVF*											*-ICVF*
L-ai		FA	-ADC					-ADC				-ADC	
		*ICVF*					*ICVF*						*-ICVF*
L-pi		FA	-ADC					-ADC*					
		*ICVF*											
ILF R			-ADC										
		*ICVF*					*ICVF*						
ILF L		FA											
		*ICVF*					ICVF*						
UF R													
		*ICVF*											
UF L													
ACR R		FA	-ADC					-ADC	-FA*	ADC			-FA
		ICVF*					*ICVF*						
ACR L		FA* *ICVF*					FA	-ADC*					

CC, Corpus callosum; SLF, Superior longitudinal fasciculus; R, right; L, left; ds, direct segment; ai, anterior indirect segment; pi, posterior indirect segment; ILF, inferior longitudinal fasciculus; UF, uncinate fasciculus; ACR, anterior corona radiata; DTI, Diffusion tensor imaging; NODDI, Neurite orientation dispersion and density imaging; FA, fractional anisotropy; ADC, apparent diffusivity coefficient; ICVF, intra-cellular volume fraction; italics, NODDI metrics; ‘-‘, the neuroimaging metric in the regression model had a negative β coefficient, indicating a negative linear relationship with the cognitive variable; *p < 0.001, all remaining cells with text are *p* < 0.05.

**Figure 2:**
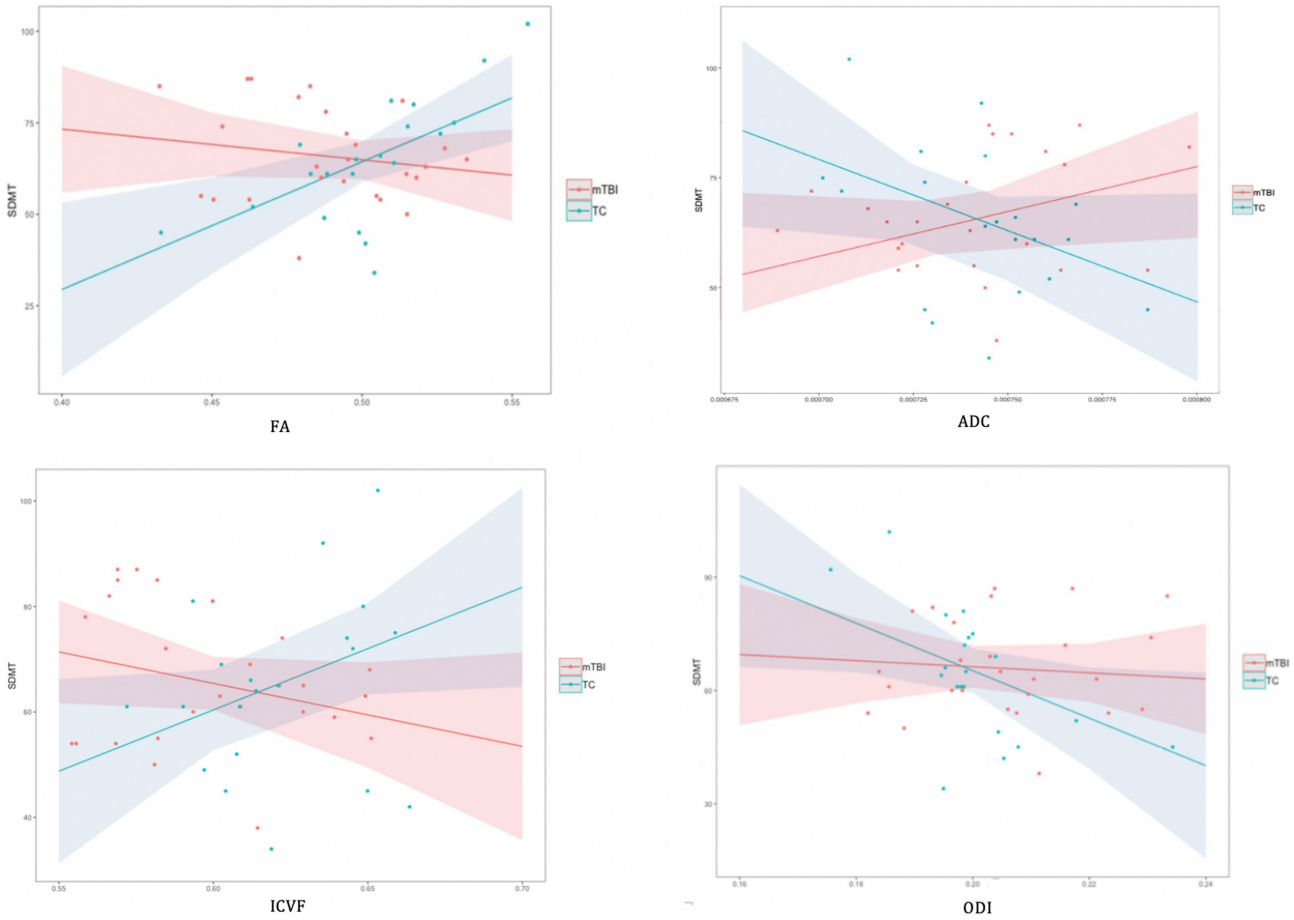
Associations between DWI metrics and SDMT performance for corpus callosum by group. In illustration of the broad pattern of findings across all significant regression analyses, the TC group showed a positive linear relationship between FA, ICVF and the cognitive variable, whereas a negative association was evident for the ADC. For the mTBI group, there were typically no significant associations between DWI metrics and cognition. There were no significant linear relationships between ODI and any cognitive variable in either group.

Overall, the results revealed a consistent pattern of neuroimaging metrics significantly predicting cognitive function across a range of WMTs for the TC group. In contrast, almost no significant predictive relationships were evident in the mTBI group. In the few instances where neuroimaging metrics did significantly predict cognition in the mTBI group, either no significant relationship was evident in the TC group, or a significant predictive relationship, in the *opposite direction,* was evident in the TC group (ADC and Memory Index in right ACR). As an exemplar illustration of this broad pattern of significant findings, Figure 2 shows the relationship between DWI metrics and SDMT performance for the CC.

For the TC group, the majority of significant results revealed a predictive relationship between imaging metrics and the SDMT and Memory Index variables. Substantially more independent predictive relationships were evident between DTI metrics and cognitive variables than were evident for NODDI metrics. FA and/or ADC independently predicted performance on the SDMT and the Memory Index in most WMTs. All significant associations between cognitive variables and FA were positive and all significant associations with ADC were negative. Of the NODDI metrics, ODI did not independently predict cognitive performance on any WMT. ICVF independently predicted cognitive performance on most WMTs, however. All significant associations between cognitive variables and ICVF were positive. The EF Index was only associated with ADC, and this occurred only for the direct segment and the anterior indirect segment of the left SLF. There were no associations between any neuroimaging metric and the SP/Attention Index.

For the mTBI group, only the SLF and the right ACR revealed a small number of significant independent predictive relationships between FA, ADC and/or ICVF and cognitive performances. In the opposite pattern to the TC group, both FA and ICVF were negatively associated with cognition and ADC was positively associated with cognition. The right ACR revealed an independent predictive relationship between DTI metrics and the Memory Index, and a small number of WMTs revealed a predictive relationship between DTI and NODDI metrics and the EF Index. There were no associations between any neuroimaging metrics and the SDMT or the SP/Attention Index.

## Discussion

Broadly consistent with the hypotheses, our findings showed a robust relationship between both DTI (FA/ADC) and NODDI (ICVF) metrics, and cognitive domains in the TC group. Specifically, the FA and ICVF metrics significantly, independently and positively predicted processing speed ability (SDMT) in the majority of WMTs; they also independently and positively predicted Memory Index performance, although these relationships were evident on a minority of WMTs. In addition, significant negative predictive relationships were found between ADC and both processing speed and the Memory Index, on the majority of WMTs. In contrast to hypotheses, for the mTBI group very few significant predictive relationships were evident between any DTI or NODDI metric and any cognitive measure on any WMT; the few significant predictive relationships that were observed were in the opposite direction to that predicted and were mostly evident for the EF Index. Also in contrast to hypotheses, neither group demonstrated any significant predictive relationships between the NODDI metric ODI, and any cognitive measure on any WMT.

These results strongly indicate that this premorbidly healthy, hospitalised TC group were performing in a manner that was commensurate with healthy performance (6, 7, 20, 21). The relationship between DWI metrics and cognition in this premorbidly healthy hospitalised mTBI group consistently contrasted that seen in the TC group, however. Wherever the TC group demonstrated a significant predictive linear relationship between DTI and/or NODDI metrics and a cognitive index, the mTBI group showed no significant predictive relationship or, in one instance, a significant relationship in the opposite direction. Further, in a small number of analyses, where no significant relationship was found for the TC group, the mTBI group did in fact reveal a significant predictive relationship.

This consistent finding of group difference in the pattern of significant relationships between DTI measures and cognition suggests that customary predictive relationships, which exist between WMT microstructure and cognitive function in premorbidly healthy hospitalised TC adults, are disrupted in premorbidly healthy hospitalised mTBI adults, 6–10 weeks after injury. Given the rigorous sample matching and the removal of influencing factors in the analyses, it is likely that this relationship disruption was specifically due to the mTBI.

The finding of contrasting group profiles between the NODDI metric, ICVF, and cognition is consistent with the single study that has looked at this previously after mTBI (25). Specifically, in the opposite pattern to the TC group, better cognitive performance by individuals with mTBI was associated with lower ICVF or lower axonal packing density (25). It was suggested this might be due to a higher ratio of astrocytes, as these have been shown to aid neuroprotection by contributing to post-traumatic tissue repair and synaptic remodelling following TBI (78).

The lack of any significant relationships between the NODDI metric, ODI, and cognition indirectly contrasts previous research, which has indicated that ODI is typically negatively correlated with FA (21) and is positively correlated with processing speed in a combined mTBI and healthy control sample (29). Given that no previous studies have reported a relationship between ODI and cognition in a pure mTBI sample, however, the present finding cannot be considered inconsistent with any previous research.

With respect to the DTI findings, a recent meta-analysis, which also examined hospitalised mixed-mechanism adults after mTBI, found a significant positive linear relationship between FA and attention, memory and executive function (9). In contrast, the present study identified *no* positive relationship between FA and cognitive function in the mTBI group on most WMTs, and even a *negative* relationship on two WMTs in the domains of memory and executive function. A possible mechanism underlying these negative relationships is that selective disruption of one WMT fibre population occurred in the presence of crossing fibres, leading to a subsequent increase in FA in the context of reduced cognitive performance (79).

The difference in DTI findings between the current and previous studies might be explained by the fact that the meta-analysis (9) collated data from different regions of interest to undertake analyses, whereas the current study looked at relationships for a substantial number of individual WMTs. In addition, most earlier studies undertook a more limited cognitive examination, included individuals that were either substantially earlier or later in the recovery process, or included individuals with moderate–severe TBI. These methodological discrepancies prevent meaningful inferential conclusions (32) regarding the discrepancy between past and present findings.

In the current study, both groups were slower than published healthy control data (77). Cognitive performances were commensurate between the groups, however, despite the relationship between cognition and WMT microstructural features (as measured by DWI metrics) being abnormal in the mTBI group. This appears to suggest that cognitive function had recovered in the mTBI group 6–10 weeks after injury to be consistent with cognitive abilities seen in general trauma patients, despite ongoing WMT structural disruptions or remodelling. It is also possible, however, that subtle group differences in cognition were not found because of the modest sample size. This is somewhat substantiated by the moderate between group effect size evident for some cognitive indices. Further research with larger sample sizes might clarify this issue.

It is noteworthy that the difference in brain-behaviour relationship between the groups occurred in the context of no group differences in post-concussion symptom reporting or quality of life. Given that symptom reporting is commonly elevated in trauma control samples (59), this does not mean that either group had recovered to premorbid levels of functioning. It does show, however, that these factors cannot explain the different pattern of relationships found between the mTBI and TC groups in this study. Future research is clearly warranted to investigate whether there is a relationship between the current pattern of findings and symptom resolution.

This study supported previous findings of CC, SLF, ILF and ACR involvement after mTBI (10–12), as the brain-behaviour relationships differed between the groups on all of these WMTs. It also indicated that the different SLF segments are comparably susceptible to an alteration in brain-behaviour relationship after mTBI. While the lack of group difference on the UF is noticeably discrepant from the remaining findings, it does not indicate that the UF was unaffected by mTBI pathology. Rather, 6–10 weeks post-injury there was no relationship between WMT structural pathology and cognition, irrespective of whether an individual suffered an mTBI or a traumatic injury without mTBI.

The present study provides an opportunity to contrast the relationship between cognition and various DWI metrics in a premorbidly healthy, hospitalised mTBI sample. Although FA and ICVF consistently demonstrated significant predictive relationships with cognition that were in the same direction, they were only concurrently significant in 30% of analyses; this is consistent with the notion that these metrics are positively correlated, but only weakly (20, 21). In contrast, whereas ODI has previously been shown to have a strong negative correlation with FA (20, 21), this relationship was not observed in the current study. Disentangling the underlying pathological mechanism for this observation is difficult, as it may relate to the complex effects of ongoing WMT microstructural damage and remodelling on the DWI signal and derived modelling metrics. Certainly, unexpected patterns of both DTI and NODDI metric changes have been reported in other mTBI studies (24, 25, 79). This finding does challenge the view, however, derived from a study using a sample of martial artists (23), that these metrics provide comparable information about underlying WMT microstructure after mTBI.

The lack of any significant predictive relationship between any DWI metric and the SP/Attention Index for either group was unexpected given previous findings of a relationship between attention and WMT microstructural organisation in healthy adults and individuals with mTBI (9, 80). The likely explanation for this was that the SP/Attention Index was not a pure measure of attention, as it included measures of attention, processing speed and mental flexibility. Although the index was created *via* a statistically robust method of creating a dimensionally coherent index (64), it resulted in an index that measured a construct other than pure attention. This prevents comparability to previous studies that utilised single measures of attention.

The primary limitation of the current study was a relatively modest sample size, which prevented investigation of interactions between WMTs and increased the likelihood of making Type II errors. To address this, we limited interpretation of our findings to overall patterns of consistent findings, as inferential conclusions are most robust when results are consistent across a range of measures (81). To reduce the likelihood of Type I error, consistent with modern statistical practise (76), rather than correcting for multiple comparisons, the findings were again interpreted with respect to overall patterns and uniformity of findings as well as considering magnitudes of difference, rather than focussing on specific *p*-values (76). Given the consistency of the pattern of significant findings in this study, there is strong evidence to support generalisability of these findings to premorbidly healthy civilian adults who are admitted to hospital with mTBI in the context of a traumatic injury.

Next, the presence of CSF partial voluming effect can confound both the DTI and NODDI metric estimates, by reducing contribution of anisotropic diffusion signal estimated from the white matter (82, 83). This is reflected by reduced FA value for the DTI model. The NODDI model has been reported to overestimate the CSF volume fraction (i.e., *fiso*) due to not accounting for compartment-specific T2 relaxation and its model parameters are usually estimated from data acquired with a single echo time (TE) (84–86). This can lead to erroneous NDI estimates in the white matter (86). Newly proposed methods, including acquiring data with multiple echo time (86), or rescaling/constrained the *fiso* modelling term (84, 85) are promising methods to adopt in future studies. Finally, tract-wise average diffusion metrics were used to represent WMT microstructural properties in this study. This has the potential of negating regional differences in microstructural properties along the WMT. To address this, alternative analyses based on obtaining diffusion metrics along the WMT profile can be adopted in future studies (87).

The clinical implications of these findings are significant. As outlined earlier, this research programme has previously reported that the present mTBI sample demonstrates WMT microstructural disorganisation relative to the TC group at this same time point post injury (13). In combination with these previously reported findings, the present study suggests that different profiles exist for cognitive vs. neurostructural resolution after mTBI. Although functional cognition appeared broadly recovered at a group level approximately 9 weeks after injury, the relationship between WMT microstructural organisation and functional cognition had significantly altered from that seen in healthy individuals. That is, functional cognition may have largely resolved not as a consequence of underlying WMT microstructural resolution, but *despite* underlying WMT microstructural disorganisation (13). This disconnection between cognitive function and WMT microstructure in recovery after mTBI raises the question of how cognition might be resolving and whether there are modifiable factors at play that could be appropriate targets for intervention.

In conclusion, this study provided strong evidence that, despite normal cognitive function at a group level, the expected predictive relationship between WMT microstructural organisation and cognitive function, which is evident in premorbidly healthy hospitalised trauma control participants, is significantly disrupted 6–10 weeks after mTBI in those who were premorbidly healthy and were hospitalised for injury. The atypical WMT structuro-functional relationship that was seen in the mTBI group was evident in the CC, the bilateral ILF and ACR, and in all SLF segments, but was essentially absent in the UF. The DTI metrics, FA and ADC, as well as the NODDI metric, ICVF, demonstrated this disruption in the domains of processing speed, memory and executive function, but the NODDI metric, ODI, did not demonstrate disruption in any domain. These findings provide robust evidence that the mTBI has caused an alteration in the way WMT microstructural organisation *relates* to cognitive function (i.e., brain-behaviour relationship), and that this alteration is evident in the post-acute period after mTBI. Future research is needed to understand if these relationships normalise over time or demonstrate lasting disruption.

## Data availability statement

The raw data supporting the conclusions of this article will be made available by the authors, without undue reservation.

## Ethics statement

The studies involving humans were approved by Alfred Hospital Human Research Ethics Committee. The studies were conducted in accordance with the local legislation and institutional requirements. The participants provided their written informed consent to participate in this study.

## Author contributions

JA: Conceptualization, Formal analysis, Funding acquisition, Methodology, Project administration, Resources, Supervision, Validation, Visualization, Writing – original draft, Writing – review & editing. LO: Conceptualization, Data curation, Formal analysis, Investigation, Methodology, Project administration, Software, Validation, Visualization, Writing – review & editing. JC: Formal analysis, Software, Writing – review & editing. JM: Conceptualization, Methodology, Writing – review & editing. MS: Conceptualization, Methodology, Resources, Supervision, Validation, Writing – review & editing, Formal analysis. JY: Conceptualization, Methodology, Resources, Supervision, Validation, Writing – review & editing, Funding acquisition, Software.

## Footnotes

^1^ http://www.mrtrix.org/

^2^ https://fsl.fmrib.ox.ac.uk/fsl
