# Supplementary material for: The relationship between cognition and white matter tract damage after mild traumatic brain injury in a premorbidly healthy, hospitalised adult cohort during the post-acute period
Source: Front Neurol. 2023 Oct 23;14:1278908. doi: 10.3389/fneur.2023.1278908 (PMC10626495; doi:10.3389/fneur.2023.1278908)
Supplement: Supplementary file 1 [file Table_1.DOCX]

**Supplementary Information**

Structure matrix of principal component analysis

Supplementary Table S1. Structure matrix for three component solution, displaying cognitive variables arranged by component

| **Cognitive variable** | **Component** | | |
| --- | --- | --- | --- |
|  | **1** | **2** | **3** |
|  | *Processing Speed/*  *High-level attention* | *Memory* | *Executive function* |
| TMT A (sec) | .740 | -.443 | -.230 |
| TMT B (sec) | .675 | -.537 | .547 |
| Stroop Dots (sec) | .821 | -.117 | -.220 |
| Digit Span total raw score | -.705 | .201 | -.484 |
| RAVLT Total Score (Trials A1–A5) | -.352 | .852 | -.316 |
| RAVLT Delayed Recall (Trial A7) | -.268 | .881 | -.121 |
| RCFT Delayed recall score | -.077 | .745 | -.069 |
| TMT Ratio score | .100 | -.154 | .865 |
| Stroop Interference Score | -.186 | -.124 | .693 |

TMT: Trail Making Test; Stroop: Victoria Stroop Test; RAVLT: Rey Auditory Verbal Learning Test; RCFT: Rey-Osterrieth Complex Figure Test

**Results**

Supplementary Table S2. Significant multiple regression analysis results for SDMT by tract for TC group

| **Tract** | **Metric** | **Bn Gp Reg Anal**  **F-statistic** | **Model R^2^** | **Ind Var** | ***B*** | ***SE B*** | **β** | **Confidence**  **Intervals** |
| --- | --- | --- | --- | --- | --- | --- | --- | --- |
| **CC** | FA | 9.183* | .772** | FA | 380.172 | 88.939 | **.599**** | **191.630 - 568.713** |
|  |  |  |  | Age | -0.024 | 0.190 | -.018 | -.426 - .378 |
|  |  |  |  | FSIQ | 0.880 | 0.197 | **.542**** | **.463 - 1.297** |
|  | ADC | 10.709* | .650** | ADC | -354982.761 | 109570.861 | **-.454*** | **-587262.610 - -122702.910** |
|  |  |  |  | Age | -0.289 | 0.191 | -.212 | -.695 - .116 |
|  |  |  |  | FSIQ | 1.064 | 0.223 | **.656**** | **.591 – 1.538** |
|  | ICVF | 6.120* | .621** | ICVF | 279.872 | 95.963 | **.426*** | **76.439 – 483.305** |
|  |  |  |  | Age | -0.426 | 0.194 | **-.313*** | **-.836 - -.015** |
|  |  |  |  | FSIQ | 1.159 | 0.239 | **.714**** | **.653 – 1.665** |
| **SLF R-ds** | ADC | 14.662** | .779** | ADC | -429491.76 | 84332.211 | **-.551**** | **-608268.060 - -250715.460** |
|  |  |  |  | Age | -0.379 | 0.148 | **-.278*** | **-.693 - -.064** |
|  |  |  |  | FSIQ | 0.950 | 0.177 | **.585**** | **.576 – 1.325** |
|  | ICVF | 7.656* | .705** | ICVF | 290.480 | 89.867 | **.449*** | **99.970 – 480.991** |
|  |  |  |  | Age | -0.539 | 0.189 | **-.396*** | **-.941 - -.138** |
|  |  |  |  | FSIQ | 0.883 | 0.224 | **.544*** | **.408 – 1.359** |
| **SLF R-ai** | FA | 7.820* | .623** | FA | 313.174 | 106.953 | **.442*** | **86.443 – 539.905** |
|  |  |  |  | Age | -0.218 | 0.207 | -.160 | -.656 - .221 |
|  |  |  |  | FSIQ | 0.970 | 0.231 | **.597**** | **.481 – 1.459** |
|  | ADC | 10.818* | .728** | ADC | -336044.314 | 94147.972 | **-.474*** | **-535629.100 - -136459.530** |
|  |  |  |  | Age | -0.331 | 0.181 | -.243 | -.715 - .053 |
|  |  |  |  | FSIQ | 1.080 | 0.215 | **.665**** | **.624 – 1.535** |
| **SLF R-pi** | ADC | 13.206** | .822** | ADC | -497258.468 | 94035.461 | **-.592**** | **-696604.740 - -297912.200** |
|  |  |  |  | Age | -0.396 | 0.145 | **-.291*** | **-.703 - -.089** |
|  |  |  |  | FSIQ | 1.308 | 0.183 | **.806**** | **.920 – 1.696** |
|  | ICVF | 6.340* | .740** | ICVF | 284.980 | 76.132 | **.497*** | **123.588 – 446.371** |
|  |  |  |  | Age | -0.533 | 0.177 | **-.391*** | **-.908 - -.157** |
|  |  |  |  | FSIQ | 1.155 | 0.214 | **.711**** | **.702 – 1.608** |
| **SLF L-ds** | FA | 6.931* | .651** | FA | 291.143 | 115.406 | **.419*** | **46.493 – 535.793** |
|  |  |  |  | Age | -0.180 | 0.226 | -.132 | -.659 - .299 |
|  |  |  |  | FSIQ | 0.937 | 0.242 | **.577**** | **.423 – 1.450** |
|  | ADC | 13.191** | .714** | ADC | -348235.720 | 103703.962 | **-.452*** | **-568078.300 - -128393.140** |
|  |  |  |  | Age | -0.367 | 0.185 | -.270 | -.758 - .024 |
|  |  |  |  | FSIQ | 0.961 | 0.219 | **.592**** | **.497 – 1.425** |
|  | ICVF | 4.511* | .653** | ICVF | 259.565 | 101.833 | **.405*** | **43.689 – 475.441** |
|  |  |  |  | Age | -0.603 | 0.213 | **-.443*** | **-1.055 - -.152** |
|  |  |  |  | FSIQ | 1.093 | 0.245 | **.673**** | **.574 – 1.612** |
| **SLF L-ai** | FA | 10.870* | .621** | FA | 241.198 | 112.343 | **.374*** | **3.042 – 479.354** |
|  |  |  |  | Age | -0.204 | 0.236 | -.150 | -.705 - .296 |
|  |  |  |  | FSIQ | 0.921 | 0.253 | **.567**** | **.384 – 1.458** |
|  | ADC | 11.993** | .719** | ADC | -387102.647 | 112785.772 | **-.462*** | **-626197.800 - -148007.490** |
|  |  |  |  | Age | -0.326 | 0.184 | -.239 | -.717 - .065 |
|  |  |  |  | FSIQ | 1.006 | 0.217 | **.620**** | **.547 – 1.466** |
|  | ICVF | 4.981* | .624* | ICVF | 232.398 | 106.064 | **.340*** | **7.554 – 457.243** |
|  |  |  |  | Age | -0.483 | 0.211 | **-.355*** | **-.931 - -.035** |
|  |  |  |  | FSIQ | 1.034 | 0.252 | **.637**** | **.501 – 1.568** |
| **SLF L-pi** | FA | 7.433* | .670** | FA | 276.387 | 99.611 | **.451*** | **65.221 – 487.552** |
|  |  |  |  | Age | -0.141 | 0.223 | -.104 | -.614 - .332 |
|  |  |  |  | FSIQ | 1.032 | 0.235 | **.636**** | **.533 – 1.531** |
|  | ADC | 17.386** | .626** | ADC | -295760.185 | 99609.158 | **-.422*** | **-506922.170 - -84598.203** |
|  |  |  |  | Age | -0.369 | 0.194 | -.271 | -.779 - .041 |
|  |  |  |  | FSIQ | 1.073 | 0.231 | **.661**** | **.583 – 1.564** |
|  | ICVF | 7.974* | .681** | ICVF | 258.488 | 88.878 | **.436*** | **70.076 – 446.900** |
|  |  |  |  | Age | -0.481 | 0.195 | **-.353*** | **-.893 - -.069** |
|  |  |  |  | FSIQ | 1.206 | 0.244 | **.743**** | **.689 – 1.722** |
| **R ILF** | ADC | 5.266* | .661** | ADC | -270486.258 | 101926.790 | **-.401*** | **-486561.400 - -54411.116** |
|  |  |  |  | Age | -0.459 | 0.200 | -.337* | -.882 - -.035 |
|  |  |  |  | FSIQ | 1.147 | 0.246 | **.707**** | **.625 – 1.669** |
|  | ICVF | 4.345* | .634** | ICVF | 235.524 | 101.728 | **.364*** | **19.871 – 451.177** |
|  |  |  |  | Age | -0.371 | 0.209 | -.272 | -.814 - .072 |
|  |  |  |  | FSIQ | 1.138 | 0.256 | **.701**** | **.594 – 1.681** |
| **L ILF** | FA | 5.683* | .640** | FA | 211.940 | 88.639 | **.386*** | **24.033 – 399.848** |
|  |  |  |  | Age | -0.237 | 0.221 | -.174 | -.706 - .232 |
|  |  |  |  | FSIQ | 0.984 | 0.245 | **.606**** | **.464 – 1.504** |
|  | ICVF | 9.154* | .734** | ICVF | 299.604 | 82.056 | **.495*** | **125.654 – 473.554** |
|  |  |  |  | Age | -0.260 | 0.183 | -.191 | -.649 - .128 |
|  |  |  |  | FSIQ | 1.146 | 0.216 | **.706**** | **.689 – 1.603** |
| **R ACR** | FA | 6.513* | .741** | FA | 277.688 | 73.830 | **.491*** | **121.175 – 434.200** |
|  |  |  |  | Age | -0.304 | 0.178 | -.223 | -.681 - .073 |
|  |  |  |  | FSIQ | 0.902 | 0.209 | **.556**** | **.459 – 1.346** |
|  | ADC | 17.686** | .705** | ADC | -284690.920 | 88039.221 | **-.465*** | **-471325.730 - -98056.109** |
|  |  |  |  | Age | -0.270 | 0.193 | -.198 | -.679 - .139 |
|  |  |  |  | FSIQ | 1.164 | 0.229 | **.717**** | **.678 – 1.650** |
|  | ICVF | 12.569** | .753** | ICVF | 294.731 | 74.545 | **.527**** | **136.702 – 452.759** |
|  |  |  |  | Age | -.542 | .173 | **-.398*** | **-.908 - -.176** |
|  |  |  |  | FSIQ | 1.245 | .214 | **.767**** | **.792 – 1.698** |
| **L ACR** | FA | 9.466* | .803** | FA | 354.440 | 72.933 | **.590**** | **199.829 – 509.050** |
|  |  |  |  | Age | -0.170 | 0.162 | -.125 | -.512 - .173 |
|  |  |  |  | FSIQ | 0.783 | 0.186 | **.482**** | **.389 – 1.178** |
|  | ICVF | 9.679* | .730** | ICVF | 335.586 | 93.310 | **.521*** | **137.778 – 533.395** |
|  |  |  |  | Age | -0.515 | 0.180 | **-.378*** | **-.896 - -.135** |
|  |  |  |  | FSIQ | 1.329 | 0.233 | **.819**** | **.834 – 1.824** |

Bn Gp Reg Anal F-statistic: F-statistic of the initial between group regression analysis, which identified statistical interactions whereby groups differed with respect to the relationship between the independent variables and cognitive variables; Model R^2^: R^2^ value for significant model, asterisk indicates level of significance for this model; Ind Var: Independent Variable; CC: Corpus callosum; SLF: Superior longitudinal fasciculus; R: right; L: left; ds: direct segment; ai: anterior indirect segment; pi: posterior indirect segment; ILF: inferior longitudinal fasciculus; ACR: anterior corona radiata; FA: fractional anisotropy; ADC: apparent diffusivity coefficient; ICVF: intra-cellular volume fraction;* p<.05; **p<.001

Supplementary Table S3. Significant multiple regression analysis results for Memory Index by tract for TC group

| **Tract** | **Metric** | **Bn Gp Reg Anal**  **F-statistic** | **Model R^2^** | **Ind Var** | ***B*** | ***SE B*** | **β** | **Confidence Intervals** |
| --- | --- | --- | --- | --- | --- | --- | --- | --- |
| **CC** | FA | 4.157* | .608** | FA | 17.370 | 6.413 | **.497*** | **3.7740-030.965** |
|  |  |  |  | Age | -0.009 | 0.014 | -.122 | -.038 - .020 |
|  |  |  |  | FSIQ | 0.040 | 0.014 | **.445*** | **.010 - .070** |
|  | ADC | 5.217* | .526* | ADC | -18415.667 | 7013.933 | **-.428*** | **-33284.541 - -3546.793** |
|  |  |  |  | Age | -0.020 | 0.012 | -.272 | -.046 - .006 |
|  |  |  |  | FSIQ | 0.049 | 0.014 | **.545*** | **.018 - .079** |
| **SLF R-ds** | ADC | 6.562* | .612** | ADC | -18405.829 | 6695.159 | **-.430*** | **-32598.933 - -4212.726** |
|  |  |  |  | Age | -0.025 | 0.012 | **-.340*** | **-.050 - -.001** |
|  |  |  |  | FSIQ | 0.043 | 0.014 | **.482*** | **.013 - .073** |
| **SLF R-ai** | ADC | 9.596* | .683** | ADC | -20042.164 | 5591.579 | **-.514*** | **-31895.781 - -8188.547** |
|  |  |  |  | Age | -.022 | .011 | -.290 | -.045 - .001 |
|  |  |  |  | FSIQ | .050 | .013 | **.563**** | **.023 - .077** |
| **SLF R-pi** | ADC | 4.887* | .624** | ADC | -21673.814 | 7527.542 | **-.469*** | **-37631.491 - -5716.137** |
|  |  |  |  | Age | -0.026 | 0.012 | **-.350*** | **-.051 - -.002** |
|  |  |  |  | FSIQ | 0.059 | 0.015 | **.657**** | **.028 - .090** |
|  | ICVF | 7.025* | .721** | ICVF | 17.747 | 4.337 | **.562**** | **8.553 – 26.940** |
|  |  |  |  | Age | -0.034 | 0.010 | **-.454*** | **-.055 - -.013** |
|  |  |  |  | FSIQ | 0.055 | 0.012 | **.618**** | **.029 - .081** |
| **SLF L-ds** | ADC | 5.261* | .618** | ADC | -18564.463 | 6585.690 | **-.438*** | **-32525.502 - -4603.423** |
|  |  |  |  | Age | -0.024 | 0.012 | -.325 | -.049 - .001 |
|  |  |  |  | FSIQ | 0.043 | 0.014 | **.485*** | **.014 - .073** |
| **SLF L-ai** | ADC | 7.907* | .681** | ADC | -23498.158 | 6609.577 | **-.509*** | **-37509.835 - -9486.482** |
|  |  |  |  | Age | -0.021 | 0.011 | -.285 | -.044 - .002 |
|  |  |  |  | FSIQ | 0.046 | 0.013 | **.514*** | **.019 - .073** |
|  | ICVF | 8.487* | .675** | ICVF | 18.885 | 5.431 | **.502*** | **7.372 – 30.397** |
|  |  |  |  | Age | -0.032 | 0.011 | **-.426*** | **-.055 - -.009** |
|  |  |  |  | FSIQ | 0.049 | 0.013 | **.545*** | **.021 - .076** |
| **SLF L-pi** | ADC | 7.461* | .752** | ADC | -22199.193 | 4866.762 | **-.576**** | **-32516.267 - -11882.119** |
|  |  |  |  | Age | -0.023 | 0.009 | **-.308*** | **-.043 - -.003** |
|  |  |  |  | FSIQ | 0.051 | 0.011 | **.575**** | **.027 - .075** |
| **R ILF** | ICVF | 4.802* | .621** | ICVF | 16.235 | 5.696 | **.457*** | **4.161 – 28.309** |
|  |  |  |  | Age | -0.024 | 0.012 | -.315 | -.048 - .001 |
|  |  |  |  | FSIQ | 0.055 | 0.014 | **.618**** | **.025 - .086** |
| **L ILF** | ICVF | 10.353* | .722** | ICVF | 18.934 | 4.615 | **.569**** | **9.151 – 28.717** |
|  |  |  |  | Age | -0.017 | 0.010 | -.226 | -.039 - .005 |
|  |  |  |  | FSIQ | 0.055 | 0.012 | **.614*** | **.029 - .081** |
| **R UF** | ICVF | 4.568* | .600* | ICVF | 14.486 | 5.527 | **.461*** | **2.770 – 26.202** |
|  |  |  |  | Age | -0.024 | 0.012 | -.326 | -.050 - .001 |
|  |  |  |  | FSIQ | 0.063 | 0.016 | **.701**** | **.029 - .096** |
| **R ACR** | ADC | 17.177** | .640** | ADC | -16398.581 | 5345.090 | **-.487*** | **-27729.665 - -5067.498** |
|  |  |  |  | Age | -0.018 | 0.012 | -.246 | -.043 - .006 |
|  |  |  |  | FSIQ | 0.055 | 0.014 | **.615**** | **.025 - .084** |
|  | ICVF | 9.110* | .571* | ICVF | 12.453 | 5.404 | **.404*** | **.997 – 23.909** |
|  |  |  |  | Age | -0.032 | 0.013 | **-.433*** | **-.059 - -.006** |
|  |  |  |  | FSIQ | 0.056 | 0.015 | **.622*** | **.023 - .088** |
| **L ACR** | FA | 4.951* | .592* | FA | 14.620 | 5.768 | **.443*** | **2.391 – 26.848** |
|  |  |  |  | Age | -0.017 | 0.013 | -.226 | -.044 - .010 |
|  |  |  |  | FSIQ | 0.036 | 0.015 | **.406*** | **.005 - .067** |
|  | ADC | 6.214* | .702** | ADC | -18083.889 | 4717.898 | **-.557**** | **-28085.386 - -8082.391** |
|  |  |  |  | Age | -0.016 | 0.011 | -.209 | -.038 - .007 |
|  |  |  |  | FSIQ | 0.056 | 0.013 | **.622*** | **.029 - .082** |

Bn Gp Reg Anal F-statistic: F-statistic of the initial between group regression analysis, which identified statistical interactions whereby groups differed with respect to the relationship between the independent variables and cognitive variables; Model R^2^: R^2^ value for significant model, asterisk indicates level of significance for this model; Ind Var: Independent Variable; CC: Corpus callosum; SLF: Superior longitudinal fasciculus; R: right; L: left; ds: direct segment; ai: anterior indirect segment; pi: posterior indirect segment; ILF: inferior longitudinal fasciculus; UF: uncinate fasciculus; ACR: anterior corona radiata; FA: fractional anisotropy; ADC: apparent diffusivity coefficient; ICVF: intra-cellular volume fraction;* p<.05; **p<.001

Supplementary Table S4. Significant multiple regression analysis results for Memory Index by tract for mTBI group

| **Tract** | **Metric** | **Bn Gp Reg Anal**  **F-statistic** | **Model R^2^** | **Ind Var** | ***B*** | ***SE B*** | **β** | **Confidence Intervals** |
| --- | --- | --- | --- | --- | --- | --- | --- | --- |
| **R ACR** | FA | 4.951* | .474* | FA | -14.309 | 3.815 | **-.603**** | **-22.220 - -6.398** |
|  |  |  |  | Age | -.026 | .009 | **-.509*** | **-.045 - -.007** |
|  |  |  |  | FSIQ | .015 | .012 | .220 | **-.010 - .040** |
|  | ADC | 17.177** | .344* | ADC | 12528.104 | 4772.754 | **.512*** | **2630.019 – 22426.190** |
|  |  |  |  | Age | -0.027 | 0.010 | **-.530*** | **-.049 - -.005** |
|  |  |  |  | FSIQ | 0.039 | 0.015 | **.586*** | **.008 - .070** |

Bn Gp Reg Anal F-statistic: F-statistic of the initial between group regression analysis, which identified statistical interactions whereby groups differed with respect to the relationship between the independent variables and cognitive variables; Model R^2^: R^2^ value for significant model, asterisk indicates level of significance for this model; Ind Var: Independent Variable; R: right; L: left; ACR: anterior corona radiata; FA: fractional anisotropy; ADC: apparent diffusivity coefficient; * p<.05; **p<.001

Supplementary Table S5. Significant multiple regression analysis results for Executive Function Index by tract for TC group

| **Tract** | **Metric** | **Bn Gp Reg Anal**  **F-statistic** | **Model R^2^** | **Ind Var** | ***B*** | ***SE B*** | **β** | **Confidence Intervals** |
| --- | --- | --- | --- | --- | --- | --- | --- | --- |
| **SLF L-ds** | ADC | 6.448* | .490* | ADC | -21177.519 | 6077.804 | **-.626*** | **-34061.888 - -8293.150** |
|  |  |  |  | Age | 0.001 | 0.011 | .023 | -.022 - .024 |
|  |  |  |  | FSIQ | 0.021 | 0.013 | .295 | -.006 - .048 |
| **SLF L-ai** | ADC | 4.581* | .439* | ADC | -24346.502 | 6420.331 | **-.661*** | **-37956.996 - -10736.008** |
|  |  |  |  | Age | 0.004 | 0.010 | .069 | -.018 - .026 |
|  |  |  |  | FSIQ | 0.024 | 0.012 | .334 | -.002 - .050 |

Bn Gp Reg Anal F-statistic: F-statistic of the initial between group regression analysis, which identified statistical interactions whereby groups differed with respect to the relationship between the independent variables and cognitive variables; Model R^2^: R^2^ value for significant model, asterisk indicates level of significance for this model; Ind Var: Independent Variable; SLF: Superior longitudinal fasciculus; L: left; ds: direct segment; ai: anterior indirect segment; ADC: apparent diffusivity coefficient; ICVF: intra-cellular volume fraction;* p<.05; **p<.001

Supplementary Table S6. Significant multiple regression analysis results for Executive Function Index by tract for mTBI group

| **Tract** | **Metric** | **Bn Gp Reg Anal**  **F-statistic** | **Model R^2^** | **Ind Var** | ***B*** | ***SE B*** | **β** | **Confidence Intervals** |
| --- | --- | --- | --- | --- | --- | --- | --- | --- |
| **SLF R-ds** | ICVF | 5.405* | .502** | ICVF | -10.646 | 4.072 | **-.434*** | **-19.091 - -2.200** |
|  |  |  |  | Age | 0.005 | 0.011 | .082 | -.018 - .028 |
|  |  |  |  | FSIQ | 0.053 | 0.014 | **.672**** | **.025 - .082** |
| **SLF R-pi** | ICVF | 11.398* | .483* | ICVF | -10.416 | 4.330 | **-.378*** | **-19.396 - -1.436** |
|  |  |  |  | Age | 0.002 | 0.011 | .025 | -.021 - .025 |
|  |  |  |  | FSIQ | 0.047 | 0.014 | **.588*** | **.017 - .076** |
| **SLF L-ds** | FA | 7.044* | .492* | FA | -14.437 | 5.748 | **-.425*** | **-26.357 - -2.517** |
|  |  |  |  | Age | -0.011 | 0.011 | -.186 | -.034 - .012 |
|  |  |  |  | FSIQ | 0.041 | 0.014 | **.521*** | **.012 - .071** |
|  | ICVF | 9.330* | .534** | ICVF | -9.805 | 3.302 | **-.444*** | **-16.652 - -2.957** |
|  |  |  |  | Age | 0.001 | 0.010 | .014 | -.021 - .022 |
|  |  |  |  | FSIQ | 0.052 | 0.013 | **.651*** | **.024 - .080** |
| **SLF L-ai** | ICVF | 7.409* | .511* | ICVF | -10.989 | 4.037 | **-.434*** | **-19.362 - -2.617** |
|  |  |  |  | Age | 0.003 | 0.011 | .043 | -.020 - .025 |
|  |  |  |  | FSIQ | 0.054 | 0.014 | **.684**** | .026 - .083 |
| **R ACR** | FA | 8.659* | .496* | FA | -11.388 | 4.459 | **-.402*** | **-20.634 - -2.141** |
|  |  |  |  | Age | -0.008 | 0.011 | -.134 | -.031 - .014 |
|  |  |  |  | FSIQ | 0.045 | 0.014 | **.559*** | **.015 - .074** |

Bn Gp Reg Anal F-statistic: F-statistic of the initial between group regression analysis, which identified statistical interactions whereby groups differed with respect to the relationship between the independent variables and cognitive variables; Model R^2^: R^2^ value for significant model, asterisk indicates level of significance for this model; Ind Var: Independent Variable; SLF: Superior longitudinal fasciculus; R: right; L: left; ds: direct segment; ai: anterior indirect segment; pi: posterior indirect segment; ACR: anterior corona radiata; FA: fractional anisotropy; ICVF: intra-cellular volume fraction;* p<.05; **p<.001
